# Degree-of-Freedom Strengthened Cascade Array for DOD-DOA Estimation in MIMO Array Systems

**DOI:** 10.3390/s18051557

**Published:** 2018-05-14

**Authors:** Bobin Yao, Zhi Dong, Weile Zhang, Wei Wang, Qisheng Wu

**Affiliations:** 1 School of Electronic and Control Engineering, Chang’an University, Xi’an 710064, China; qshwu@chd.edu.cn; 2 School of Highway, Chang’an University, Xi’an 710064, China; zdong@chd.edu.cn; 3 School of Electronic and Information Engineering, Xi’an Jiaotong University, Xi’an 710049, China; wlzhang1984@gmail.com (W.Z.); AmyWang1986@163.com (W.W.)

**Keywords:** difference co-array, cascade array, angle estimation, weighted subspace fitting, multiple input multiple output systems

## Abstract

In spatial spectrum estimation, difference co-array can provide extra degrees-of-freedom (DOFs) for promoting parameter identifiability and parameter estimation accuracy. For the sake of acquiring as more DOFs as possible with a given number of physical sensors, we herein design a novel sensor array geometry named cascade array. This structure is generated by systematically connecting a uniform linear array (ULA) and a non-uniform linear array, and can provide more DOFs than some exist array structures but less than the upper-bound indicated by minimum redundant array (MRA). We further apply this cascade array into multiple input multiple output (MIMO) array systems, and propose a novel joint direction of departure (DOD) and direction of arrival (DOA) estimation algorithm, which is based on a reduced-dimensional weighted subspace fitting technique. The algorithm is angle auto-paired and computationally efficient. Theoretical analysis and numerical simulations prove the advantages and effectiveness of the proposed array structure and the related algorithm.

## 1. Introduction

In the past few decades, research on the theory and algorithms of estimating direction of arrival (DOA) in array processing usually focused on the overdetermined system model with uniform linear array (ULA) configuration. A basic piece of knowledge is that the number of sources that can be resolved by the MUSIC algorithm is N−1 if adopting a *N*-element ULA [1,2]. However, many scenarios imply an underdetermined system model in which the number of unknown sources may be actually more than the number of physical sensors. To solve such a challenging problem, most techniques are based on creating the effect of a equivalent array with many more virtual elements than actual array through suitable spatial-domain, time-domain or frequency-domain sampling strategies.

The earliest pioneering studies, by exploiting the possible element arrangements, such as space tapered arrays and fractal-based quasi random array [3,4,5], are designed for yielding reasonably low sidelobe level; and, recently, the random array has been integrated into a hardware prototype to achieve sub-Nyquist sampling in spatial and spectral domains [6]. One of the most popular virtual arrays is that of synthetic aperture radar (SAR), where the larger artificial aperture is created by means of the motion of radar antennas [7]. In wide-band array signal processing, all sub-band signals are usually aligned into one single frequency to antagonize the coherence of signals, which can be viewed as a special virtual array that takes advantage of the frequency diversity [8,9]. In recent years, one of the most popular strategies that can provide the ability of resolving more sources than sensors is the so-called co-array. For example, in the work of [10], the quasi-stationary signals are considered to construct a virtual ULA for improving the parameter identifiability. Basically, the concept of co-array can be categorized into sum co-array and difference co-array. The first one usually appears in some active sensing systems such as co-located MIMO radar [11], whereas the second one appears in the passive one such as traditional direction finding system or bistatic MIMO radar [12,13,14,15,16,17,18,19].

Generally speaking, for an *N*-element array, both the sum co-array and difference co-array can create up to $O(N^{2})$ elements, depending on the geometry of the physical sensor array. Theoretically, minimum redundancy array (MRA) [20] is the optimum linear geometry, which can create the maximum possible number of elements in difference co-array without any missing elements in between, but retains some unwilling repeated elements. However, the MRA with a given *N* has no closed-form expression either for its structure or the achievable degrees-of-freedom (DOFs), and hence its geometry is not analytically tractable. By combining two or more ULAs with increasing inter-sensor spacing, Ref. [13] designs a new array structure named a nested array, which is capable of providing a dramatic increase in DOFs and hence can resolve more sources than the actual number of sensors. Later, the coprime sensing array [14] and its generalized versions [15] are designed for efficient sampling of multidimensional signals, which can be directly applied to the aforementioned underdetermined model.

Although the above array geometries/structures all can provide $O(N^{2})$ DOFs, there still exist quantitative differences. Furthermore, such difference determines the capability of resolving more sources than sensors. By comparison, in the sense of difference co-array with a given number of physical sensors, MRA sets an upper-bound of DOFs; the DOFs provided by nested array [13] is greater than the one of the coprime arrays [14], and both configurations are inferior to the coprime array with displaced subarrays (CADiS) [15]. However, the CADiS cannot stay consecutive in the whole co-array because it has holes. Actually, promoting the potential DOFs closely to the upper-bound is still an open challenge problem, the kernel of which relies on a more effective design for sensors’ positions.

In addition, when we apply the concept of difference co-array into the multiple input multiple output (MIMO) array systems such as MIMO radar or some wireless communication systems, the other key point is the parameter estimation algorithm with respect to direction-of-arrival (DOA) or carrier frequency. For example, Refs. [13,14,21,22,23,24] adopt multiple signal classification (MUSIC) spectrum searching to estimate DOA information, and Refs. [25,26,27,28,29] discuss algorithms with super resolution from the perspective of sparsity-based recovery. Unfortunately, however, the above algorithms or tensor-based algorithms [19], Refs. [30,31] will bring huge computational complexity in practice if more than one-dimensional parameters are considered. There exist non-spectrum-searching choices, e.g., the estimation of signal parameters via rotational invariance techniques (ESPRIT) algorithm [32] and MUSIC-rooting algorithm, which can decrease the calculation amount to some extent, but they are usually at the cost of reducing angle estimation accuracy.

In this paper, motivated by the need for efficiently solving the aforementioned problems, a series of work is developed to improve the exist weaknesses. The main contributions are three-fold:

1. A new array structure named cascade array is designed, which is essentially composed of one ULA and one non-uniform linear array. Through theoretical analysis, the difference co-array of the designed optimal cascade array is hole-free and can provide more DOFs than some state-of-the-art sensor array structures. That is to say, it manifests a strengthened resolving capability.
2. We then apply the cascade array into bistatic MIMO array systems to achieve joint direction-of-departure (DOD) and DOA estimation for multiple targets localization. Furthermore, by parameterizing the orthogonal projector onto the null space of the equivalent joint steering matrix, a novel algorithm based on a reduced-dimensional weighted subspace fitting technique is proposed.
3. The proposed algorithm transforms a two-dimensional estimation problem into a one-dimensional one. To do so, the DOD information can be acquired by MODE rooting [33,34], and the auto-paired DOA information can be extracted from the estimated receiving array manifold. The proposed algorithm avoids exhausted spectrum searching or tensor decomposition [19,34,35], thus it is computationally efficient.

The rest of this paper is organized as follows: in Section 2, we introduce the cascade array and its optimization. The application in MIMO array systems and related two-dimensional difference co-array are presented in Section 3. In Section 4, we provide an effective joint DOD and DOA estimation algorithm. Numerical simulations are shown in Section 5, followed by the conclusions in Section 6.

**Notation**: ${\left(\cdot \right)}^{*}$, ${\left(\cdot \right)}^{T}$, ${\left(\cdot \right)}^{H}$, ${\left(\cdot \right)}^{†}$ denote the complex conjugate, transpose, Hermitian transpose, pseudo-inverse, respectively. Symbol ⊗ represents a Kronecker product and symbol ⊙ is Khatri–Rao product, which is a column-wise Kronecker product. $I_{M}$ is a M×M identity matrix and 0 symbolizes zero matrix. $A^{\left(m\right)}$ is a submatrix of A formed by its last *m* rows. Operator ∠[·] serves to get the phase. In addition, as a shorthand notation, the addition between a set and a scalar is defined as S±b={a±b|∀a∈S} and the difference set between $S_{1}$ and $S_{2}$ is given by $\mathrm{Diff}\left(S_{1},S_{2}\right)=\left\{a-b\;|\;a\in S_{1},b\in S_{1}\right\}$.

## 2. Cascade Array

In this section, we will introduce the detailed structure of our designed cascade array, and then make a DOF comparison with other types of array configuration. For convenience, we first introduce the concept of difference co-array and its closed relationship with the Khatri–Rao product of two steering vectors [10] and [13], which plays an important role in the follow-up content.

### 2.1. Difference Co-Array

Given a sensor position set $P=\{p_{i},1\leq i\leq N\}$, the difference co-array with respect to set P can be created by

(1) $$P_{d}=\left\{\bar{p}\;|\;\bar{p}=p_{i}-p_{k},p_{i}\in P,p_{k}\in P\right\},$$

which means that each element in $P_{d}$ represents a virtual sensor.

It is worth mentioning that there exist some repeated values and/or ‘holes’ in this new set, which is completely determined by the array geometry. The number of DOFs with respect to a real set P is the cardinality of its difference co-array set. If defining $|P_{d}|$ be the cardinality of set $P_{d}$, i.e., the number of distinct elements, as we know, $|P_{d}|=2N-1$ for a ULA, whereas that for MRA, nested array, coprime array, CADiS, the coprime array with compressed inter-element spacing (CACIS) [15] is $O(N^{2})$.

To establish a relationship between difference co-array and sensor array manifold $C\left(\theta \right)\in C^{N\times K}$, let us consider the Khatri–Rao product, $\tilde{C}\left(\theta \right)=C^{*}\left(\theta \right)⊙C\left(\theta \right)$. If defining *p*-th column of C(θ) be $c(\theta_{p})$, then correspondingly the *p*-th column of $\tilde{C}\left(\theta \right)$ is given by

(2) $$\tilde{c}\left(\theta_{p}\right)=c^{*}\left(\theta_{p}\right)⊗c\left(\theta_{p}\right)={\left[c_{1}^{*}c^{T}\left(\theta_{p}\right),c_{2}^{*}c^{T}\left(\theta_{p}\right),\cdots ,c_{N}^{*}c^{T}\left(\theta_{p}\right)\right]}^{T},$$

where $c_{i}$ is the *i*-th element of vector $c(\theta_{p})$. It can be seen that there is a direct connection between $\tilde{c}\left(\theta_{p}\right)$ and $P_{d}$ since the elements of $\tilde{c}\left(\theta_{p}\right)$ are given by

(3) $${\left[\tilde{c}\right]}_{l}=e^{j\pi (p_{i}-p_{k})\sin\theta },\;l=\left(m-1\right)N+n.$$

Obviously, each row of $\tilde{c}\left(\theta_{p}\right)$ has a one-to-one correspondence with the element in $P_{d}$, and therefore $\tilde{C}\left(\theta \right)$ implies the manifold of a virtual array whose sensors are located at the positions indicated by set $P_{d}$.

A more effective array geometry means that it should yield as many distinct elements as possible in concrete natural numbers, which characterizes larger DOFs or a strong capability of resolving more sources than sensors; and simultaneously it should have no vacancy or holes, which characterizes a virtual ULA structure and also a free usage of traditional angle estimation algorithms. Both requirements will also be a criterion in the following designing and comparison.

### 2.2. Cascade Array Design

**Figure 1:**
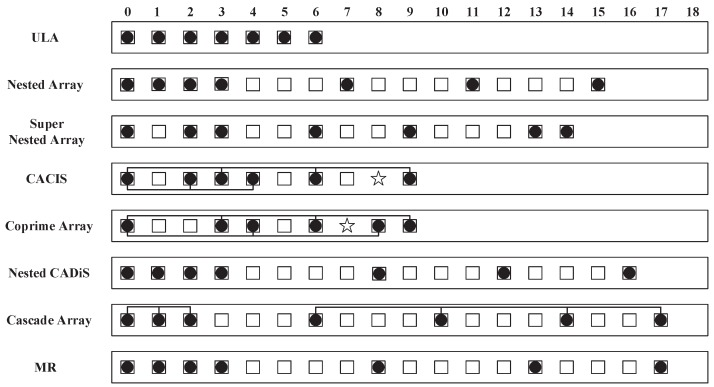
An example with total N=7 sensors for different array configurations: Bullets stand for physical sensors, boxes for difference coarray in the nonnegative part and stars for holes.

The kernel of designing array geometry is to determine and optimize the array elements’ positions. As we know, the nested array or coprime array is still rooted in uniform linear geometry with different element-spacing. Taking a two-level nested array [13], for example, as the array structure is shown in Figure 1, the second level ULA has a spacing that is determined by the number of the first level ULA. The biggest advantages of such structure are of easily designing and of easily figuring out the specific DOFs. However, the pursuit of more DOFs cannot be confined by that uniform feature, e.g., MRA, which is a typical example that can achieve the largest DOFs through non-uniform features. The cascade array in this research essentially borrows the non-uniform feature and meanwhile inherits the feature of nested array.

Assuming the total number of sensors is $N=N_{1}+N_{2}$ and a basic spacing unit *d* is set as half-wavelength, i.e., d=λ/2, where λ is the carrier wavelength. Our cascade array can be partitioned into two parts. Just like the nested array, the first level array is still a $N_{1}$-element ULA with one spacing unit, but, on the contrary, the second level array is a $N_{2}$-element non-uniform linear array.

Compared with nested array, there are mainly two modifications. On the one hand, we enlarge the spacing between two arrays up to $\left(N_{1}+1\right)$ spacing unit, the purpose of which is to strengthen the DOF, but it will incur holes; therefore, on the other hand, we further shorten the spacing of the last two sensors in the second level array, which can mend that defect. In a word, the designed cascade array is described by a precise definition, i.e.,

**Definition** **1.** *Assume $N_{1}$ and $N_{2}$ are integers satisfying $N_{1}\geq 1$ and $N_{2}\geq 2$. Second-order cascade array is specified by the integers set L, defined by*

(4) $$L=L^{1}\cup L^{2}\cup L^{3},$$

*where each subset is given as below:*

$$\begin{array}{ccc} L^{1} & = & \{0,1,2,\cdots ,N_{1}-1\},\\ L^{2} & = & \left\{0,\left(N_{1}+1\right),\cdots ,\left(N_{2}-2\right)\left(N_{1}+1\right)\right\}+2N_{1},\\ L^{3} & = & \left\{\left(N_{2}-2\right)\left(N_{1}+1\right)+N_{1}\right\}+2N_{1}, \end{array}$$

And its difference co-array set is

(5) $$P_{d}^{L}=\mathrm{Diff}\left(L,L\right)=\left\{\bar{p}\;|\;\bar{p}=p_{i}-p_{k},p_{i}\in L,p_{k}\in L\right\}.$$

In set L, $L^{1}$ denotes the ULA and $L^{2}\cup L^{3}$ denotes the non-uniform one. The typical configurations for different array geometry with total N=7 sensors is shown in Figure 1. In this example, we make a graphical illustration for the designed cascade array, ULA, nested array [13], super nested array [30], CACIS [15], coprime array [14], nested CADiS (it is an optimum solution of CADiS under the requirement for highest number of consecutive lags) [15] and MR array [20]. We can see that the designed cascade array can generate more DOFs than other six array geometries and attain the same performance as MR array.

To completely exploit the DOFs of the cascade array, we summarize the properties of $P_{d}^{L}$ in the following theorem.

**Theorem** **1.** The second-order cascade array in Definition 1 is a kind of restricted array [13], i.e, its difference co-array is hole-free; and it can provide DOFs with the number of $2N_{2}\left(N_{1}+1\right)+2N_{1}-3$.

**Proof.**  The strict and detailed derivations are shown in Appendix A. ☐

According to the above theorem, we can further reach a new corollary with respect to the problem that optimizes the distribution of sensors in the configuration of cascade array by finding $N_{1}$ and $N_{2}$ to maximize the total DOFs under a fixed number of sensors.

**Corollary** **1.** *Given N physical sensors, e.g., $N=N_{1}+N_{2}$, the optimal choices of $N_{1}$ for the 1st level array and $N_{2}$ for the 2nd level array in the designed cascade array configuration are verified as*
**N****Optimal**
$N_{1}$
**and**
$N_{2}$**The Number of DOFs**Even$N_{1}=N_{2}=\frac{N}{2}$$\frac{N^{2}}{2}+2N-3$Odd$N_{1}=\frac{N-1}{2},\;N_{2}=\frac{N+1}{2}$$\frac{N^{2}-1}{2}+2N-3$

**Proof.**  The solution is easily obtained by utilizing arithmetic mean-geometric mean (AM-GM) inequality, and we skip it for simplicity. ☐

The detailed comparison with respect to the DOFs, parameter identifiability and estimation accuracy of the designed cascade array will be introduced in Section 5.

## 3. MIMO Array Systems with Cascade Array

In this section, we will apply the aforementioned results into MIMO array systems to achieve higher accuracy of parameters estimation, and mainly focus on the problem of targets localization, i.e., the joint direction of departure (DOD) and direction of arrival (DOA) estimation.

### 3.1. Data Model

We herein consider a MIMO array systems with cascade array configuration at both transmitting and receiving end, in which *M* and *N* antenna elements are arranged, respectively. The whole system is frequency synchronization or fully calibrated [36]. It is also assumed that there are *K* targets, and the output baseband signal of the matched filters at the receiving end can be written as [18,19]

(6) $$y\left(t\right)=\left[b\left(\phi_{1}\right)⊗a\left(\theta_{1}\right),\cdots ,b\left(\phi_{K}\right)⊗a\left(\theta_{K}\right)\right]h\left(t\right)+w\left(t\right),$$

where the transmitting steering vector $a(\theta_{k})$ and the receiving steering vector $b(\phi_{k})$, for k=1,2,⋯,K, are assumed to be unchanged during a coherent processing interval (CPI), and $\theta_{k},\phi_{k}$ are the DOD and DOA of the *k*-th target, respectively. We herein adopt a Swerling I target model, so the signal vector $h\left(t\right)={\left[\gamma_{1}\left(t\right),\cdots ,\gamma_{K}\left(t\right)\right]}^{T}$ relies on the Doppler frequency $f_{dk}$ and the radar cross section (RCS) coefficient $\beta_{k}$, i.e., then $\gamma_{k}\left(t\right)=\beta_{k}e^{j2\pi f_{dk}\left(t-1\right)}$. Note that $a\left(\theta_{k}\right)={\left[e^{j\pi l_{1}\sin\theta_{k}},\cdots ,e^{j\pi l_{M}\sin\theta_{k}}\right]}^{T}$ and $b\left(\phi_{k}\right)={\left[e^{j\pi p_{1}\sin\phi_{k}},\cdots ,e^{j\pi p_{N}\sin\phi_{k}}\right]}^{T}$ are mutilated Vandermonde vectors, where ${\left\{l_{m}\right\}}_{m=1}^{M}$ and ${\left\{p_{n}\right\}}_{N=1}^{N}$ is the antennas’ position defined by the cascade array configuration. w(t) is the additive zero-mean Gaussian noise with covariance $\sigma_{n}^{2}$.

Defining $A=\left[a\left(\theta_{1}\right),\cdots ,a\left(\theta_{K}\right)\right]\in C^{M\times K}$, $B=\left[b\left(\phi_{1}\right),\cdots ,b\left(\phi_{K}\right)\right]\in C^{N\times K}$, and after collecting *Q* consecutive pulses, Label (6) can be rewritten by the following compact form:

(7) Y=(B⊙A)S+W,

where Y=[y(1),y(2),⋯,y(Q)], S=[h(1),h(2),⋯,h(Q)] and W=[w(1),w(2),⋯,w(Q)].

### 3.2. Two-Dimensional Difference Co-Array

Assuming the Doppler frequencies of all *K* targets are all different and also well separated. The covariance matrix of the received data y(t) is given by

(8) $$R=E\left[y\left(t\right)y^{H}\left(t\right)\right]=\left(B⊙A\right)\Lambda {\left(B⊙A\right)}^{H}+\sigma_{n}^{2}I_{MN}.$$

Due to the *K* target reflected signals in ${\left\{\gamma_{k}\left(t\right)\right\}}_{k=1}^{K}$ are sinusoidal signals with different frequencies and attenuation coefficients, they are temporally uncorrelated. If sampling them by a sufficient large snapshot rate (at least twice the maximum Doppler frequency), then the signal autocorrelation matrix Λ is diagonal, and Λ=diag[p] with $p={\left[\beta_{1}^{2},\cdots ,\beta_{K}^{2}\right]}^{T}$.

We vectorize R to get the following vector:

(9) $$r=vec\left(R\right)=\left[{\left(B⊙A\right)}^{*}⊙\left(B⊙A\right)\right]p+\sigma_{n}^{2}1,$$

where vec(·) denotes the vectorizing operator. $1={\left[e_{1}^{T},\cdots ,e_{MN}^{T}\right]}^{T}$ with $e_{i}$ being a vector of all zeros except a 1 at the *i*-th position.

Compared with one-dimensional scenario, it is a little more complicated when achieving difference co-array under the two-dimensional scenario, such as Label (9). Hence, we introduce the following lemma:

**Lemma** **1.** *Consider a auxiliary matrix defined by $\Pi =I_{N}⊗\Gamma ⊗I_{M}$, where*

$$\Gamma ={\left[\begin{array}{cccc} \Delta \\ 0_{M\times 1} & \Delta \\ \vdots & \vdots & \ddots \\ 0_{M\times 1} & 0_{M\times 1} & \ldots & \Delta \end{array}\right]}^{MN\times MN}\;\;\;\;and\;\;\;\;\Delta ={\left[\begin{array}{cccc} 1\\ 0_{1\times N} & 1\\ \vdots & \vdots & \ddots \\ 0_{1\times N} & 0_{1\times N} & \ldots & 1 \end{array}\right]}^{M\times [N(M-1)+1];}$$

*then, the following result will hold:*

(10) $$\Pi \left[{\left(B⊙A\right)}^{*}⊙\left(B⊙A\right)\right]=\left(B^{*}⊙B\right)⊙\left(A^{*}⊙A\right).$$

The above conclusion utilizes some properties of the Khatri–Rao product of multiple matrices, and one can refer to [37] for more detail.

According to the lemma above, after left-multiplying the permutation matrix Π on Label (9), we can acquire a new constructed data with the following form:

(11) $$\bar{r}=\Pi r=\left[\left(B^{*}⊙B\right)⊙\left(A^{*}⊙A\right)\right]p+\sigma_{n}^{2}\Pi 1.$$

The vector $\bar{r}$ can be viewed as one-pulse baseband observationof a virtual MIMO radar with equivalent transmitting steering matrix $\left(A^{*}⊙A\right)$ and equivalent receiving steering matrix $\left(B^{*}⊙B\right)$ in a deterministic noise environment.

We define $\tilde{A}$ and $\tilde{B}$ as the virtual transmitting and the virtual receiving steering matrices after deleting the redundant items (It can be achieved by deleting the corresponding row observations in $\bar{r}$). Therefore, Label (11) can be further rewritten as:

(12) $$\tilde{r}=\left[\tilde{B}⊙\tilde{A}\right]p+\sigma_{n}^{2}e.$$

The *k*-th column of $\tilde{B}$ and $\tilde{A}$, respectively, is represented as:

(13) $$\begin{array}{ccc} \tilde{b}\left(\phi_{k}\right) & = & {\left[e^{-j\pi \bar{N}sin\phi_{k}},\cdots ,1,\cdots ,e^{j\pi \bar{N}sin\phi_{k}}\right]}^{T}, \end{array}$$

(14) $$\begin{array}{ccc} \tilde{a}\left(\theta_{k}\right) & = & {\left[e^{-j\pi \bar{M}sin\theta_{k}},\cdots ,1,\cdots ,e^{j\pi \bar{M}sin\theta_{k}}\right]}^{T}, \end{array}$$

where $\bar{M}=l_{M},\bar{N}=p_{N}$. e is a zero column vector, except a 1 in the middle.

### 3.3. Identifiability Analysis

Now, we have to discuss the parameter identifiability for data model Label (12), i.e., the maximal number of targets that can be uniquely identified without noise because it is a prerequisite for a well-posed parameter estimation. Generally speaking, the identifiability for a given data model is acting like a theoretical upper bound. We herein only consider the angle estimation algorithms based on signal/noise subspace technique.

Actually, most signal subspace based algorithms for parameter estimating require no rank deficiency in the observation matrix; however, we can see that the data $\tilde{r}$ is rank one. Therefore, we have to make a spatial smoothing before signal subspace decomposition.

Define the following $\left(\bar{M}+1\right)\left(\bar{N}+1\right)\times \left(\bar{M}+1\right)\left(\bar{N}+1\right)$ selection operator

(15) $$\Xi_{n,m}=\left[0_{\left(\bar{N}+1\right)\times \left(\bar{N}+1-n\right)}\;I_{\left(\bar{N}+1\right)}\;0_{\left(\bar{N}+1\right)\times \left(n-1\right)}\right]⊗\left[0_{\left(\bar{M}+1\right)\times \left(\bar{M}+1-m\right)}\;I_{\bar{M}+1}\;0_{\left(\bar{M}+1\right)\times \left(m-1\right)}\right],$$

where $1\leq n\leq \bar{N}+1$, $1\leq m\leq \bar{M}+1$. If we stack the selected observation vector $\tilde{r}$ as follows, we have

(16) $$R\left(\tilde{r}\right)=\left[\Xi_{1,1}\tilde{r}\;\cdots \;\Xi_{1,\bar{M}+1}\tilde{r}\;\;\Xi_{2,1}\tilde{r}\cdots \;\Xi_{2,\bar{M}+1}\tilde{r}\;\cdots \;\Xi_{\bar{N}+1,\bar{M}+1}\tilde{r}\right].$$

Then, the above two-dimensional virtual spatial smoothing operation yields

(17) $$\bar{Y}=R\left(\tilde{r}\right)=\left[B⊙A\right]\bar{S}+\sigma_{n}^{2}I_{\left(\bar{M}+1\right)\left(\bar{N}+1\right)}=\bar{A}\bar{S}+\sigma_{n}^{2}I_{\left(\bar{M}+1\right)\left(\bar{N}+1\right),}$$

where the equivalent signal matrix is

(18) $$\bar{S}=\Lambda {\left[B⊙A\right]}^{H}.$$

For convenience, we let $B={\tilde{B}}^{\left(\bar{N}+1\right)}$, $A={\tilde{A}}^{\left(\bar{M}+1\right)}$, and their *k*-th column is denoted by $\bar{b}\left(\phi_{k}\right)$ and $\bar{a}\left(\theta_{k}\right)$, respectively.

**Theorem** **2.** *For a MIMO array systems with cascade array configuration, see Label (7), if we perform two-dimensional difference co-array by Lemma 1 to get Label (12) and further to get Label (17), the parameter set $\left(\theta_{k},\phi_{k}\right)$, k=1,2,⋯,K, can be uniquely identified if*

(19) $$\left\{ \begin{array}{cc} K\leq \left(\bar{N}+1\right)\left(\bar{M}+1\right)-1, & for\;2D-MUSIC,\\ K\leq \bar{N}\left(\bar{M}+1\right), & for\;2D-ESPRIT, \end{array}\right.$$

*where the parameters’ set is drawn from a continuous distribution with respect to the Lebesgue measure in $L^{2K}$, L:=[−π/2,π/2].*

**Proof.**  Suppose the parameters are all drawn from a continuous distribution. To view in retrospect the model described in Label (17) and illuminating this by the almost surely full column rank of the Khatri–Rao product of two Vandermonde matrices [37,38,39], i.e., $rank\left[C_{1}^{M_{1}\times K}⊙C_{2}^{M_{2}\times K}\right]=\min\left(M_{1}M_{2},K\right)$, we know that, providing $\left(\bar{N}+1\right)\left(\bar{M}+1\right)\geq K$, $\bar{A}$ and ${\bar{A}}^{H}$ are all almost surely *K* rank. The spectrum searching algorithms, such as two-dimensional MUSIC algorithm: $\phi ,\theta \in [-\frac{\pi }{2},\frac{\pi }{2}]$

(20) $$P\left(\phi ,\theta \right)=\frac{1}{{\left[\bar{b}\left(\phi \right)⊗\bar{a}\left(\theta \right)\right]}^{H}U_{0}U_{0}^{H}\left[\bar{b}\left(\phi \right)⊗\bar{a}\left(\theta \right)\right],}$$

where $U_{0}$ denotes the noise subspace of $\bar{Y}$, and it usually must be at least one-dimension; therefore, $K\leq \left(\bar{N}+1\right)\left(\bar{M}+1\right)-1$ can guarantee the parameter identifiability with probability one. In addition, in the ESPRIT algorithm [18], partitioning the signal subspace $U_{s}$ for calculating steering matrix has to require $K\leq \bar{N}\left(\bar{M}+1\right)$. ☐

**Remark** **1.** *In some very special cases, it has $rank(\tilde{A})<K$ even though $K\leq \left(\bar{N}+1\right)\left(\bar{M}+1\right)-1$; however, the theorem tells us that such cases are measure-zero events, that is to say, $\tilde{A}$ is almost surely full column rank.*

Previous results on the maximum upper bound of the parameter identifiability such as MUSIC-like or rotational invariance algorithms are MN−1 when the uniform array configuration is used. Due to the non-uniform feature in the cascade array, it usually satisfies $\bar{M}>M$ and $\bar{N}>N$ so that a much stronger identifiability is acquired. It is worth mentioning that, although the upper-bound of parameter identifiability is derived under the noise-less case, it still works under the limited snapshot number and low signal-to-noise ratio (SNR) case.

## 4. Joint DOD and DOA Estimation

In this section, we mainly focus on introducing a new computational efficiency joint DOD and DOA estimating algorithm, which is based on a subspace fitting technique.

### 4.1. Weighted Subspace Fitting

As we know, an asymptotically statistically efficient estimation under a large number of snapshots or high signal-to-noise ratio (SNR) can be obtained by minimizing a weighted subspace fitting [1]:

(21) $$F\left(\theta ,\phi \right)=\mathrm{tr}\left\{P_{B⊙A}^{⊥}U_{s}WU_{s}^{H}\right\},$$

where $\theta ={\left[\theta_{1},\theta_{2},\cdots ,\theta_{K}\right]}^{T}$ and $\phi ={\left[\phi_{1},\phi_{2},\cdots ,\phi_{K}\right]}^{T}$; the diagonal weighted matrix

(22) $$W={\left(\Sigma_{s}-{\hat{\sigma }}_{n}^{2}I\right)}^{2}\Sigma_{s}^{-1}$$

with the estimated noise variance

(23) $${\hat{\sigma }}_{n}^{2}=\frac{1}{\left(\bar{M}+1\right)\left(\bar{N}+1\right)-K}\sum_{i=K+1}^{\left(\bar{M}+1\right)\left(\bar{N}+1\right)}\lambda_{i}$$

$U_{s}$ is the signal subspace of $\bar{Y}$, and its corresponding eigenvalues ${\left\{\lambda_{i}\right\}}_{i=1}^{K}$ forms $\Sigma_{s}$.

In addition, $P_{B⊙A}^{⊥}$ in Label (21) stands for the orthogonal projector onto the null space of ${\left(B⊙A\right)}^{H}$, the expression of which is given by,

(24) $$P_{B⊙A}^{⊥}=I-\left(B⊙A\right){\left[{\left(B⊙A\right)}^{H}\left(B⊙A\right)\right]}^{-1}{\left(B⊙A\right)}^{H}.$$

For minimizing function Label (21), two-dimensional searching in the whole angle-domain is a very direct approach, but it is of computational inefficiency. We herein adopt a MODE-like algorithm that makes use of polynomial rooting. The MODE algorithm was originally proposed in [40] to deal with one-dimensional parameters estimation. For our two-dimensional case, the biggest difficult relies on how to parameterize $P_{B⊙A}^{⊥}$ because B and A are coupling. Therefore, we have to introduce an substitute.

To begin, we first try to parameterize the above projector by a coefficient vectors $b={\left[b_{0},b_{1},\cdots ,b_{K}\right]}^{T}$. These coefficients construct a polynomial with the following form:

(25) $$\sum_{i=0}^{K}b_{i}z^{K-i}=b_{0}\prod_{i=1}^{K}\left(z-e^{j\pi \sin\phi_{i}}\right),\;\;b_{0}\neq 0.$$

If we introduce the following set

(26) $$L=\left\{\left\{b_{i}\right\}|C\left(z\right)=\sum_{i=0}^{K}b_{i}z^{K-i}\neq 0\;\;\;\mathrm{for}\;\;\;|z|\neq 1\right\},$$

it can be seen that the mapping from $\{\phi_{i}\}\in R$ to $\{b_{i}\}\in L$ is one-to-one providing we eliminate the non-uniqueness implied by the introduction of $b_{0}\neq 1$.

Let $G_{b}\in C^{\left(\bar{N}+1\right)\times \left(\bar{N}+1-K\right)}$ be the following Toeplitz matrix:

(27) $$G_{b}^{H}=\left[\begin{array}{cccccc} b_{K} & \cdots & b_{1} & b_{0} & \cdots & 0\\ 0 & \ddots & \ddots & \ddots & \ddots & 0\\ 0 & \cdots & b_{K} & \cdots & b_{1} & b_{0} \end{array}\right].$$

It is observed that $\mathrm{rank}\left\{G_{b}\right\}=\bar{N}+1-K$, and

(28) $$G_{b}^{H}B=0.$$

Based on the above relation, we can conclude the following theorem that can be utilized to estimate all the DOA information.

**Theorem** **3.** *Let the columns of $G_{b}$ span the null space of $B^{H}$, and if defining $G=G_{b}⊗I_{\bar{M}+1}$, then $\mathrm{span}\left\{G\right\}\subset \mathrm{span}\left\{U_{0}\right\}$.*

**Proof.**  According to Label (28), the columns of G span a column space that can guarantee the following result:

$$G^{H}\left(B⊙A\right)=\left(G_{b}^{H}B\right)⊙A=0.$$

On the other hand, if considering the rank of matrix G, it is shown that

(29) $$\mathrm{rank}\left\{G\right\}=\mathrm{rank}\left\{G_{b}\right\}\times \mathrm{rank}\left\{I_{M}\right\}=\left(\bar{N}+1-K\right)\left(\bar{M}+1\right).$$

As we know, the rank of noise subspace $U_{0}$ is $\mathrm{rank}\left\{U_{0}\right\}=\left(\bar{M}+1\right)\left(\bar{N}+1\right)-K$. Obviously, if and only if $\bar{M}\geq 1$, then

(30) $$\mathrm{rank}\left\{U_{0}\right\}>\mathrm{rank}\left\{G\right\}.$$

Such result directly demonstrates that the column space spanned by the columns of G is included in the null space spanned by $U_{0}$. This completes the proof. ☐

Therefore, we can construct a projection matrix

(31) $$P_{G}=G{\left(G^{H}G\right)}^{-1}G^{H}=P_{G_{b}}⊗I_{\bar{M}+1},$$

where $P_{G_{b}}=G_{b}{\left(G_{b}^{H}G_{b}\right)}^{-1}G_{b}^{H}$ ; then, we substitute $P_{G}$ for $P_{B⊙A}^{⊥}$ in Label (21), and, correspondingly, a new objective function that needs to be minimized is reformulated as

(32) $$F\left(b\right)=\mathrm{tr}\left\{\left(P_{G_{b}}⊗I_{M}\right)U_{s}WU_{s}^{H}\right\}.$$

If comparing function F(θ,ϕ) with function F(b), we can see that: on the one hand, the original two-dimensional optimization problem is transformed into an one-dimensional problem, that is to say, it greatly decreases the complexity; on the other hand, the projector matrix $P_{B⊙A}^{⊥}$ is parameterized by a series of coefficients. However, such operation combined with the subsequent angle estimation algorithm inevitably incurs performance loss if comparing with the two-dimensional spectrum searching method for optimizing F(θ,ϕ). The related analysis and comparison for such performance loss will be reserved for future work.

### 4.2. Angle Estimation

In order to implement the minimization of Label (32) more conveniently, we define

(33) $$\bar{U}=U_{s}WU_{s}^{H}=[\begin{array}{ccc} {\bar{U}}_{11} & \cdots & {\bar{U}}_{1N}\\ \vdots & \ddots & \vdots \\ {\bar{U}}_{N1} & \cdots & {\bar{U}}_{NN} \end{array}],$$

where ${\bar{U}}_{uv}$ is a $\left(\bar{M}+1\right)\times \left(\bar{M}+1\right)$ matrix, $u,v=1,\cdots ,\bar{N}+1$. Furthermore, we can get

(34) $$\hat{b}=\arg\min\mathrm{tr}\left\{P_{G_{b}}\left[\begin{array}{ccc} \mathrm{tr}({\bar{U}}_{11}) & \cdots & \mathrm{tr}({\bar{U}}_{1N})\\ \vdots & \ddots & \vdots \\ \mathrm{tr}({\bar{U}}_{N1}) & \cdots & \mathrm{tr}({\bar{U}}_{NN}) \end{array}\right]\right\}.$$

In addition, we also exert such constraints on the unknown parameters ${\left\{b_{i}\right\}}_{i=1}^{\bar{N}+1}$, i.e., $b_{i}=b_{\bar{N}+1-i}^{*}$. The detailed discussion with respect to the above constraint and procedures for minimizing the above quadratic function can be found in [33,41].

Once we get $\hat{b}$, the angular phase of the roots of the estimated polynomial in Label (25) will give the DOA information of all targets, i.e., ${\left\{{\hat{\phi }}_{i}\right\}}_{i=1}^{K}$.

For DOD information, the following method is adopted. We convert the two-dimensional MUSIC algorithm, see Label (20), into two optimization problems [42,43]

(35) $$\begin{array}{c} \max_{\phi }\;e^{T}E^{-1}\left(\phi \right)e, \end{array}$$

(36) $$\begin{array}{c} \min_{\theta }\;\bar{a}{\left(\theta \right)}^{H}E\left(\phi \right)\bar{a}\left(\theta \right),\;s.t.\;e^{T}\bar{a}\left(\theta \right)=1, \end{array}$$

where $E\left(\phi \right)={\left[\bar{b}\left(\phi \right)⊗I_{\bar{M}+1}\right]}^{H}U_{0}{U_{0}}^{H}\left[\bar{b}\left(\phi \right)⊗I_{\bar{M}+1}\right]$ and $e={\left[1,0,\cdots ,0\right]}^{T}$.

Due to the fact that we have obtained DOA information ${\left\{\phi_{k}\right\}}_{k=1}^{K}$ through coefficient vector $\hat{b}$, the auto-paired DOD information $\theta_{k}$ can be drawn from the estimated transmit steering vectors, i.e.,

(37) $$\hat{\bar{a}\left(\theta_{k}\right)}=\frac{E{\left({\hat{\phi }}_{k}\right)}^{-1}e}{e^{T}E{\left({\hat{\phi }}_{k}\right)}^{-1}e},$$

which can be achieved easily by utilizing Lagrange multiplier method to Label (36), and consequently

(38) $${\hat{\theta }}_{k}=\arcsin\left\{\frac{1}{\pi \bar{M}}\sum_{\bar{m}=1}^{\bar{M}}∠\frac{{\bar{a}}_{k}\left[\bar{m}+1\right]}{{\bar{a}}_{k}\left[\bar{m}\right]}\right\},$$

where ${\bar{a}}_{k}\left[\bar{m}\right]$ denotes the $\bar{m}$-th element of $\hat{\bar{a}\left(\theta_{k}\right)}$.

### 4.3. Computational Complexity

We now analyze the computational complexity of the above algorithm. By neglecting some trivial operations, Table 1 lists the number of flops required in major steps. Here, the SVD is assumed to be performed by the Golub–Reinsch SVD algorithm [44]. For convenience, let $\tilde{M}=\bar{M}+1$ and $\tilde{N}=\bar{N}+1$.

In Table 1, parameter ζ denotes the iterative number for optimization of MODE algorithm, which is usually selected as 4 [33]. Compared with the proposed algorithm, tensor-based algorithms [34,35] have to pay out large computational cost for calculating high-order singular value decomposition; and they also have to afford two-times of computational burden because two quadratic functions need separately minimization in the procedures of performing MODE algorithm. Consequently, the angle pairing is inevitable. In addition, the proposed algorithm requires more flops than two-dimensional ESPRIT algorithm (it requires $QM^{2}N^{2}+{\tilde{M}}^{3}{\tilde{N}}^{3}+3\bar{N}\tilde{M}K^{2}+26K^{3}+\tilde{M}\tilde{N}K^{2}$ flops) and much less than the two-dimensional MUSIC algorithm.

## 5. Numerical Simulation

In this section, we will fully demonstrate the effectiveness of the designed array configuration and the proposed angle estimation algorithm by Monte Carlo simulations. In the following examples, the Doppler frequency $f_{dk}$ is generated by $f_{dk}=\left(2\pi \upsilon_{k}T_{p}\right)/\lambda$, where $\upsilon_{k}$ is the target velocity, $T_{p}=5\times 10^{-6}$ is the pulse duration in seconds, and $\lambda =3\times 10^{8}/f_{c}$ with $f_{c}=3$ GHz. The average root mean square error (RMSE) vs. signal-noise-ratio (SNR) is used as our performance assessment, where the SNR is defined according to Lable (7):

$$\mathrm{SNR}=10\log_{10}\frac{{∥\left(B⊙A\right)H∥}^{2}}{MN\sigma_{n}^{2}},$$

and the average root-mean-square error (RMSE) is defined as

$$\left(1/K\right)\sum_{k=1}^{K}\sqrt{E\left[{\left({\hat{\theta }}_{k}-\theta_{k}\right)}^{2}+{\left({\hat{\phi }}_{k}-\phi_{k}\right)}^{2}\right].}$$

### 5.1. DOF Comparison

**Figure 2:**
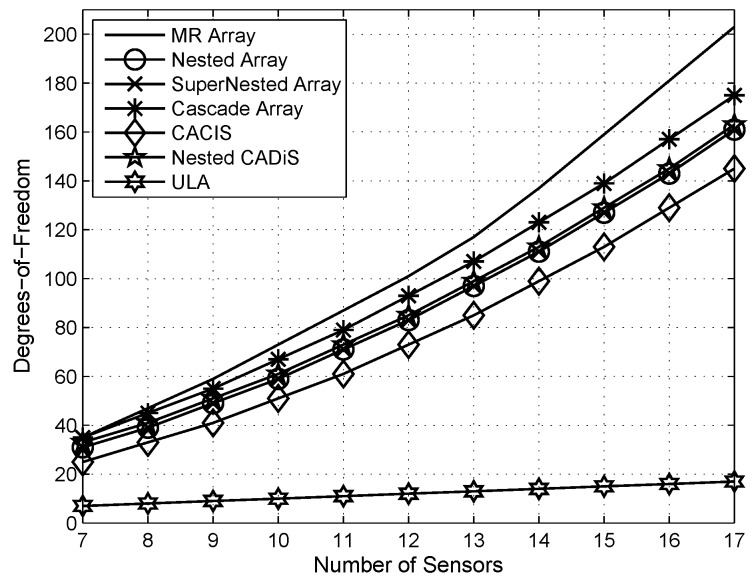
The number of DOFs comparison with different array configurations.

We first compare different array configurations from the perspective of maximum consecutive DOFs. As the results shown in Figure 2, we can conclude that the designed cascade array can generate more DOFs than other exist arrays such as ULA, nested array [13], super nested array [16], CACIS and nested CADiS [15]; and reaches a closed performance to the DOFs upper bound of the MR array [20].

### 5.2. Identifiability Performance for Cascade Array

In order to sufficiently examine the identifiability performance of the designed cascade array under the underdetermined and normal scenarios, we present a one-dimensional DOA case and MIMO DOD-DOA case through the following examples.

**Figure 3:**
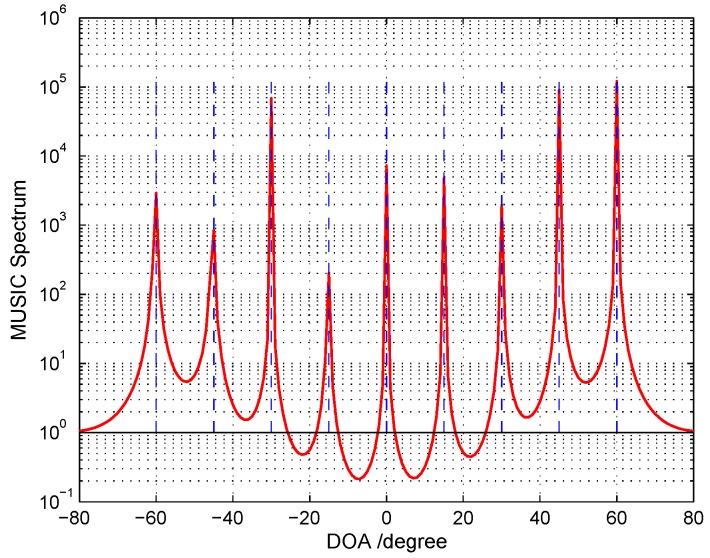
MUSIC spectrum in one-dimensional underdetermined case: nine uncorrelated signals impinge on 5-element cascade array, SNR =10 dB and Q=500.

**1D underdetermined scenario:** We arrange such a underdetermined scenario that nine far-field narrow-band uncorrelated signals impinge an array with only N=5 sensors from direction-of-arrival (DOA) of $\left\{-60^{\circ },-45^{\circ },-30^{\circ },-15^{\circ },0^{\circ },15^{\circ },30^{\circ },45^{\circ },60^{\circ }\right\}$. The sources are modeled as random Gaussian processes and all with equal power. The signal-to-noise ratio is 10 dB and the number of snapshots is 500. From Figure 3, we can see that all sources can be resolved sufficiently well by cascade array: {0,1,4,7,9}. However, other array geometries cannot provide enough DOFs to fulfill the requirement of such spectrum estimation, e.g., the nested array: {0,1,2,5,8}; the CACIS: {0,1,2} and {0,3,6}; the ULA: {0,1,2,3,4}; the super nested array cannot be constructed due to its requirement for n≥7. In addition, the nested CADiS: {0,1,2,6,9} can perform the same resolving ability as our cascade array under that underdetermined case, but its identifiability becomes smaller than the cascade array when sensor number *n* increases (see Figure 2).

**Figure 4:**
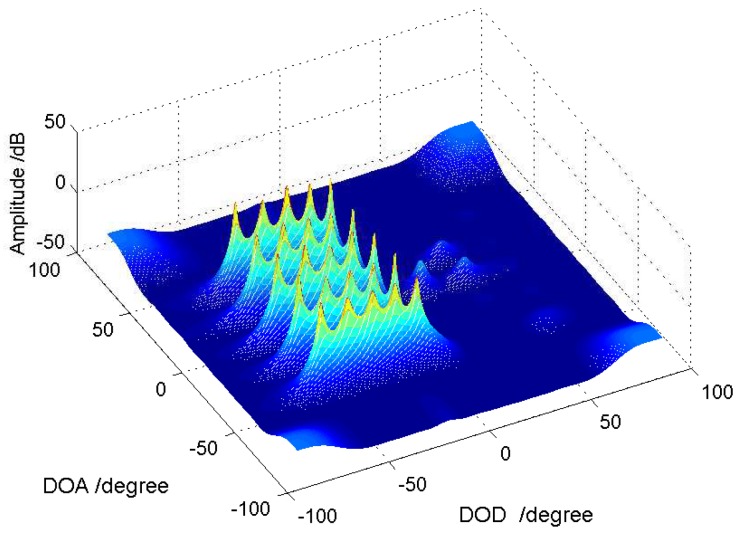
MUSIC spectrum in two-dimensional underdetermined case: 25 uncorrelated targets’ reflected signals in M=N=5 MIMO array system (adopting cascade array configuration), SNR =10 dB and Q=500.

**2D underdetermined scenario:** We consider an underdetermined MIMO array systems with M=N=5 sensors. For traditional MIMO array with ULA configuration, according to the almost surely full column rank of the Khatri–Rao product of two Vandermonde matrixes, the upper bound of the parameter identifiability is MN−1=24; however, the virtual MIMO array based on the cascade array is $\left(\bar{N}+1\right)\left(\bar{M}+1\right)-1=99$. We set 25 targets with reflected signals coming from two-dimensional angle-domain uniformly, the RCS coefficients of which are set as ${\left\{\beta_{k}\right\}}_{k=1}^{25}=1$. The SNR is 10 dB and the number of snapshots is 500. The MUSIC spectrum P(ϕ,θ) we used is given by Label (20). From Figure 4, we can see that, under such two-dimensional underdetermined scenario, the designed cascade array can distinguish all 30 targets successfully.

**Figure 5:**
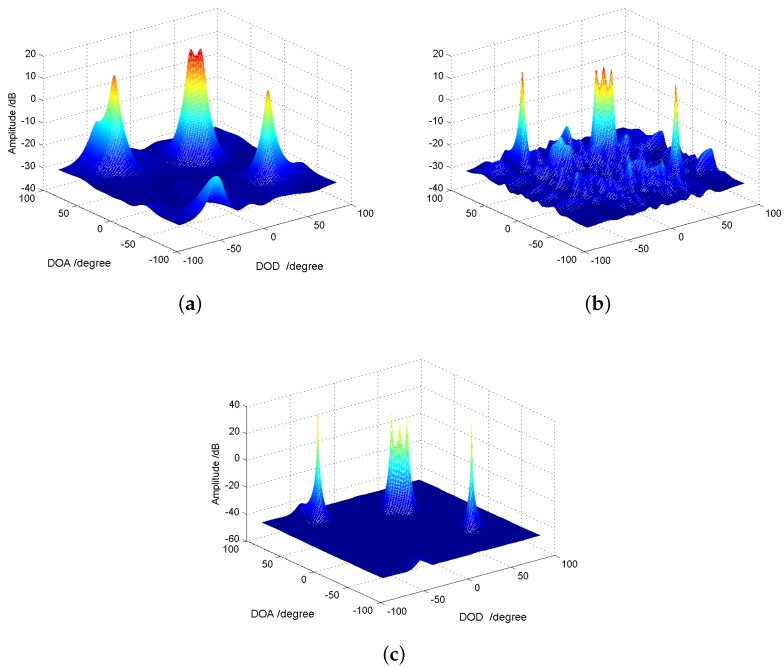
MUSIC spectrum in two-dimensional normal case, SNR =8 dB, Q=100, K=5 targets: (**a**) ULA with M=N=5; (**b**) random array with M=N=5; (**c**) cascade array with M=N=5.

**2D normal scenario:** Furthermore, Figure 5 plots the two-dimension MUSIC spectrum under a normal scenario: K=5 targets with $\theta =\{40^{\circ },35^{\circ },30^{\circ },-40^{\circ },65^{\circ }\}$ and $\phi =\{20^{\circ },25^{\circ },30^{\circ },50^{\circ },-45^{\circ }\}$, i.e., for three closely spaced targets and two targets widely spaced from the others. The other parameters are $\left\{\beta_{k}\right\}=\left\{1,1,1,1,1\right\}$, Q=50 and SNR =8 dB. In this simulation, we also consider the random array configuration. According to [28,29], the total aperture for both transmitting and receiving array is set as 10 half-wavelengths. To ensure the aperture length, we fixed the location of two elements at the extremities and placed the rest of the elements uniformly at random in between. In this single realization of random array, the transmitting array is {0,2.7581,3.1378,4.7333,10} and the receiving array is {0,0.6562,2.8234,7.8127,10}. From the simulation results, one can see that: (1) for M=N=5 ULA configuration, the two-dimensional MUSIC spectrum cannot give a accurate localization for that three closely spaced targets because that three peaks merged, while the other targets are well located; (2) for M=N=5 random array configuration, the distinguishing performance goes better; (3) for the cascade array configuration, it significantly improves the spatial resolution not only in the closely spaced targets becoming distinguishable, but also in the sidelobe suppression.

### 5.3. RMSE Performance for Joint DOD and DOA Estimation

**Figure 6:**
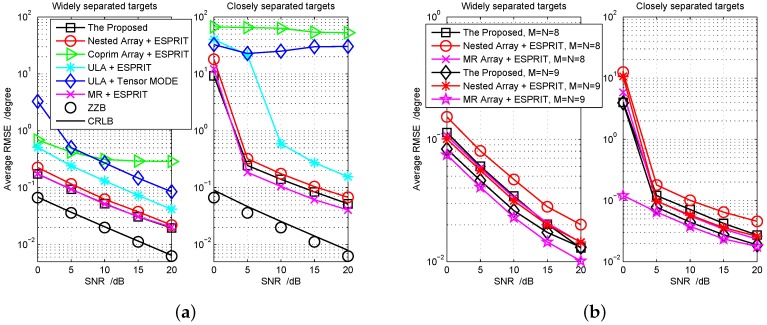
Performance comparison with different array configurations and algorithms for widely separated targets and closely separated targets respectively, Q=200, K=6 targets: (**a**) the first simulation example, M=N=7; (**b**) the second simulation example.

In Figure 6, we compare the performance of some existing algorithms, including the tensor MODE algorithm with M=N=8 uniform linear array configuration [34], 2D-ESPRIT algorithm with M=N=7 nested array configuration [13] (Note that the super nested array [16] has the same DOF as the nested array when the number of antenna is larger than 7, so we herein just consider the nested array configuration), 2D-ESPRIT algorithm [19] with M=N=8 coprime array configuration [14] (It consists of two ULAs with five sensors and 2×2 sensors, respectively, and their first sensors are of coincidence) and the proposed algorithm with M=N=7 cascade array configuration. In addition, we also compare our earlier proposed method [18], which is based on the MR array. We consider two cases, i.e., K=6 widely separated targets: $\left(\theta ,\phi \right)=\{\left(-80^{\circ },70^{\circ }\right),\;\left(-60^{\circ },10^{\circ }\right),\;\left(-40^{\circ },50^{\circ }\right),\;\left(-20^{\circ },-30^{\circ }\right),$$\left(0^{\circ },-10^{\circ }\right),\left(20^{\circ },30^{\circ }\right)\}$ with the RCS coefficients $\left\{\beta_{k}\right\}=\left\{1,\;0.9,\;0.8,\;0.7,\;0.6,\;0.5\right\}$ and closely separated targets: $\left(\theta ,\phi \right)=\{\left(-80^{\circ },70^{\circ }\right),\;\left(-75^{\circ },65^{\circ }\right),\;\left(-40^{\circ },50^{\circ }\right),\;\left(-35^{\circ },45^{\circ }\right),$ $\left(0^{\circ },-10^{\circ }\right),\left(5^{\circ },-15^{\circ }\right)\}$ with the RCS coefficients $\left\{\beta_{k}\right\}=\left\{1,\;0.85,\;0.7,\;0.55,\;0.4,\;0.25\right\}$. We fix the number of pulses Q=200 for both cases. For the average RMSE performance evaluation, the global bound—Ziv–Zakai bound (ZZB) [45,46] and the local bound, Cramér–Rao lower bound (CRLB) [47]—are all provided as benchmarks.

**Figure 7:**
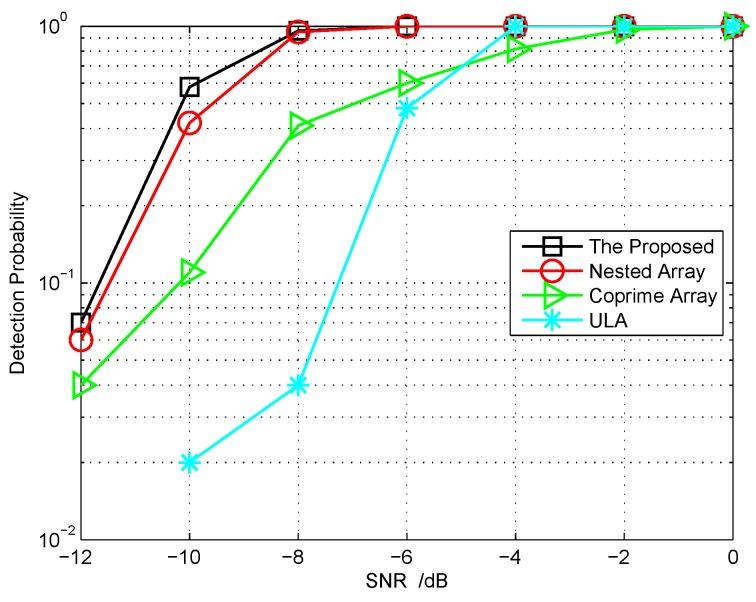
The detection probability of target number comparison with different array configurations.

From the first simulation results in Figure 6, we can see that the designed cascade array with the proposed algorithm performs the same as the method based on the MR array [18] for the widely separated targets, and performs a little worse for the closely separated targets. However, it outperforms other types of array configurations. The major manifestation lies in the lower average RMSE of DOD-DOA estimation can be attained. Furthermore, in the second example, the performance gap between the nested array and cascade array when M=N=8 is larger than the one when M=N=7. Such advantage benefits from the strengthened DOF generated by the designed cascade array. According to Section 2, for transmitting array or receiving array, the DOF difference of the co-arrays between cascade array and nested array is 4 when M=N=7 (The cascade array provides 35 DOFs and the nested array provides 31 DOFs); then, it increases to 6 when M=N=8 (The cascade array provides 45 DOFs and the nested array provides 39 DOFs). On the other hand, such advantage cannot persist in the case with many more antennas. For instance, seeing the case of M=N=9 in the second example, the performance improvement will become smaller and smaller with the SNR increasing. The major reason behind this phenomenon is that there exists a model error during the procedures of achieving co-array, i.e., the matrix Λ of Label (8) is approximately diagonal in estimating the actual correlation matrix R so that it plays a dominant adverse role in the high SNR region. In the second example, undoubtedly, the method based on the MR array [18] is optimal because it has an upper-bound of DOFs. For the RMSE lower bound, in calculating ZZB, the prior distribution of the DOD and DOA (sinθ and sinϕ) are uniform on the unit disc with covariance $\frac{1}{4}I$ [46]. For the widely separated targets scenario in Figure 6a, the ZZB merges with the CRLB, but it becomes a loose bound under the closely separated targets scenario. The reason is that the ZZB depends on the prior distribution of parameters rather than the parameters so that both scenarios share the same ZZB; however, the CRLB varies with different scenarios. In addition, we do not plot the performance at much lower SNR. The reason comes from Figure 7, that is to say, the detection of targets number, which is a prerequisite for angle estimation, cannot guarantee being absolutely right when the SNR is very low.

It is worth mentioning that the above result does not mean the ZZB is inferior to the CRLB. As we know [45,46,48], the performance of angle estimation is characterized by the presence of distinct SNR regions. Besides the asymptotic region we consider in this simulation, there still exist the a priori performance region (characterized by small snapshots and/or lower SNR) and an additional ambiguity region (or transition region). The ZZB can provide a much tighter bound in all regions and even can accurately identify the SNR thresholds, whereas the CRLB is not qualified.

### 5.4. Other Related Performance

**Targets number Estimation:** In the aforementioned simulation examples, the targets number is assumed to be known as a prior condition. In Figure 7, we try to consider the targets number detection performance under several different sensor array configurations. In this simulation, the second order statistic of the eigenvalues (SORTE) algorithm [49] is adopted, which is an eigenvalue-based strategy. The all parameters setting is the same as the previous example, that is to say, the targets number K=6 that needs to be detected. The detection probability (DP), $N_{K}/N_{tol}$, versus SNR is depicted, in which the $N_{tol}$ is the total trials and $N_{K}$ is the number of times that *K* is successfully detected. From the simulation results, we can see that the designed cascade array configuration outperforms the nested array, coprime array and ULA with the same number of antennas.

**Figure 8:**
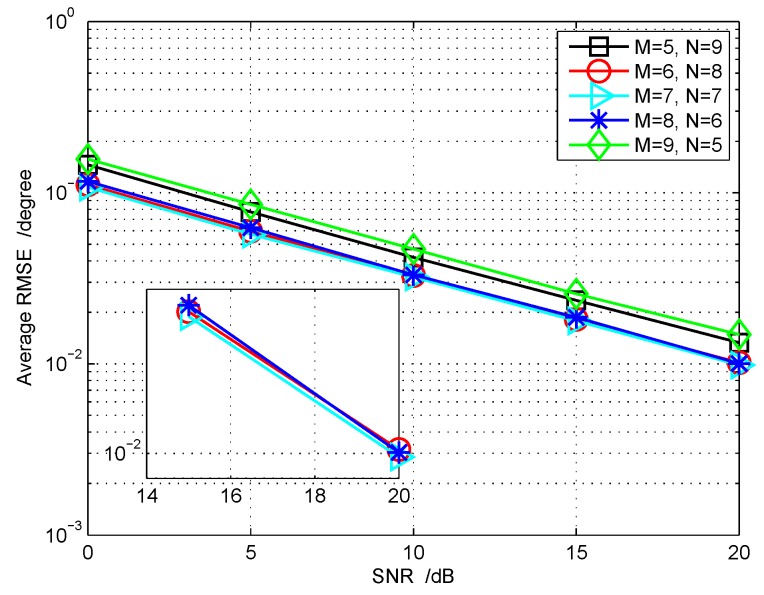
Performance comparison with different combinations of (*M*, *N*): M+N=14.

**Figure 9:**
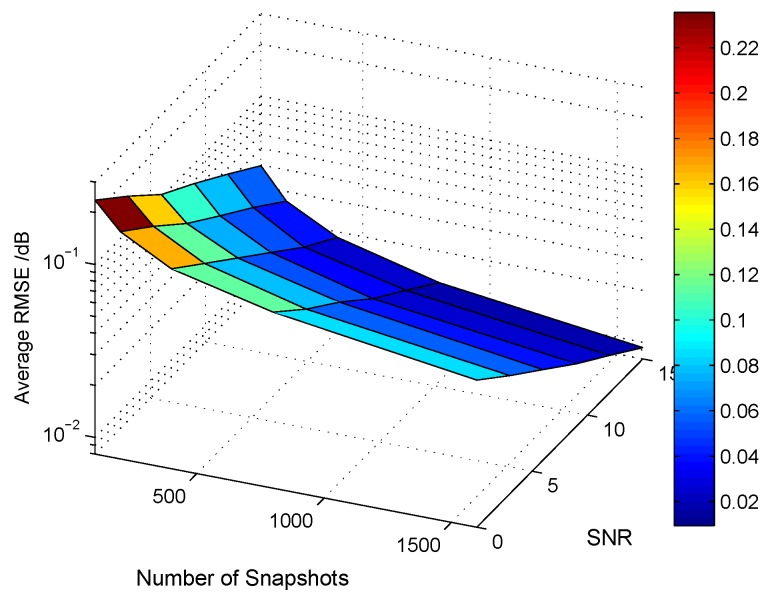
The average RMSE performance with a different number of snapshots.

**Performance with different M,N and different snapshot number:** In the following, we first fix the total number of transmitting-receiving antennas, i.e., M+N=14, to examine which combination of {M,N} can provide the optimal average RMSE performance. The angle setting is $\left(\theta ,\phi \right)=\{\left(45^{\circ },50^{\circ }\right),\;\left(35^{\circ },30^{\circ }\right),\;\left(15^{\circ },10^{\circ }\right),\;\left(-15^{\circ },-10^{\circ }\right),$ $\left(-25^{\circ },-30^{\circ })\right\}$. From Figure 8, we can see that the simulation results under M=N=7 have the least average RMSE. In addition, we also consider the angle estimation performance versus different number of pulses, as is shown in Figure 9, which demonstrates that the increase of pulse number can also improve the target’s localization accuracy. It is worth mentioning that a large pulse number will make matrix Λ much more diagonally dominant; consequently, it can diminish to some extent the model error.

## 6. Conclusions

In this paper, we mainly focus on two problems in the multiple input multiple output array systems: one is to design a new linear array structure, which can provide more degrees of freedom than some existing arrays from the perspective of different co-arrays; the other is to propose a new targets’ localization algorithm with respect to DOA and DOD, which contains only one-time polynomial rooting and two-dimension angle estimation auto-pairing. We not only analyze systematically the optimal configuration of the designed cascade array under a given number of antennas and the maximal parameter identifiability when applying it into the MIMO array systems, but also provide some simulation proofs from the angle estimation accuracy and the target’s number detection probability to verify the effectiveness and advantages of the designed array structure and the proposed targets’ localization algorithm.

In the future work, we will mainly focus on three aspects. First, more efficient angle estimation algorithms should be devised because there exists an obvious performance gap with the Cramér–Rao lower bound; second, the cascade array or other array geometries should be optimized or designed to generate more DOFs because there exists a DOF gap with the MR array; finally, the applications to other different scenarios with the proposed algorithm and array geometry are also worth considering. 

## Figures and Tables

**Table 1:** Computational complexity analysis.

Operation	Dimension Size	Required Flops
Eigenvalue Decompositon of $\bar{Y}$: $U_{s}$ and $\Sigma_{s}$	MN×Q	$QM^{2}N^{2}+{\tilde{M}}^{3}{\tilde{N}}^{3}$
Matrix $\bar{U}$ Partition in Label (34)	$\tilde{M}\tilde{N}\times \tilde{M}\tilde{N}$	$\frac{1}{2}\tilde{M}{\tilde{N}}^{2}$
Minimization of F(b)	$\tilde{N}\times \tilde{N}$	$\frac{1}{2}\zeta K\left(\tilde{N}-K\right)\left(20K^{2}+33K+17\right)$
$\bar{a}\left(\theta \right)$ Estimation	$\tilde{M}\times \tilde{M}$	${\tilde{M}}^{3}$
Total flops		$O\{{\tilde{M}}^{3}{\tilde{N}}^{3}\}$

## Appendix A. The DOF of Cascade Co-Array $P_{d}^{L}$

The statement that a kind of array configuration is a restricted array is equivalent to the argument: for every *i* ranging from $-N_{2}\left(N_{1}+1\right)-N_{1}+2$ to $N_{2}\left(N_{1}+1\right)+N_{1}-2$, there exists one physical sensor locating at *i* or at least one pair of physical sensors with sensor separation *i*. Nevertheless, it is not necessary to inspect all possible *i*s because of the two properties:

1. If *i* belongs to the co-array, then its counterpart, i.e., −i, is also in the same co-array.
2. i=0 is in the co-array that is determined by the first physical sensor or by the self-difference of any one of the physical sensors.

Hence, it is reasonable to evaluate the scenario that $1\leq i\leq N_{2}\left(N_{1}+1\right)+N_{1}-2$. We consider the following cases:

If $1\leq i\leq N_{1}-1$, it comes from $L^{1}$ and its self-difference set $\mathrm{Diff}(L^{1},L^{1})$.

If $i=\left(q-1\right)\left(N_{1}+1\right)+N_{1},q=1,2,\cdots ,N_{2}-1$, we should check set

$$\begin{array}{c} \;\;\mathrm{Diff}(L^{3},L^{2})\\ =\left\{\left(N_{2}-2\right)\left(N_{1}+1\right)+N_{1}\right\}+2N_{1}-\left\{0,\left(N_{1}+1\right),\cdots ,\left(N_{2}-2\right)\left(N_{1}+1\right)\right\}-2N_{1}\\ =\{\left(N_{2}-2\right)\left(N_{1}+1\right)+N_{1},\left(N_{2}-3\right)\left(N_{1}+1\right)+N_{1},\cdots ,N_{1}\}. \end{array}$$

If $q\left(N_{1}+1\right)\leq i\leq q\left(N_{1}+1\right)+N_{1}-1,q=1,2,\cdots ,N_{2}-1$, we should turn to calculate set

$$\begin{array}{c} \;\;\mathrm{Diff}(L^{2},L^{1})\\ =\left\{0,\left(N_{1}+1\right),2\left(N_{1}+1\right),\cdots ,\left(N_{2}-2\right)\left(N_{1}+1\right)\right\}+2N_{1}-\left\{0,1,2,\cdots ,N_{1}-1\right\}\\ =\left\{\left(N_{1}+1\right),2\left(N_{1}+1\right),\cdots ,\left(N_{2}-1\right)\left(N_{1}+1\right)\right\}+\left(N_{1}-1\right)-\left\{0,1,2,\cdots ,N_{1}-1\right\}\\ =\left[q\left(N_{1}+1\right)+\left(N_{1}-1\right)\right]-\left\{0,1,2,\cdots ,N_{1}-1\right\}\\ =\{q\left(N_{1}+1\right)+\left(N_{1}-1\right),q\left(N_{1}+1\right)+\left(N_{1}-2\right),\cdots ,q\left(N_{1}+1\right)\}, \end{array}$$

where $q=1,2,\cdots ,N_{2}-1$.

If $\left(N_{2}-1\right)\left(N_{1}+1\right)+N_{1}\leq i\leq \left(N_{2}-1\right)\left(N_{1}+1\right)+2N_{1}-1$, i.e., $N_{2}\left(N_{1}+1\right)-1\leq i\leq N_{2}\left(N_{1}+1\right)+N_{1}-2$, we can consider set

$$\begin{array}{c} \;\;\mathrm{Diff}(L^{3},L^{1})\\ =\left\{\left(N_{2}-2\right)\left(N_{1}+1\right)+N_{1}\right\}+2N_{1}-\left\{0,1,2,\cdots ,N_{1}-1\right\}\\ =\{\left(N_{2}-1\right)\left(N_{1}+1\right)+2N_{1}-1,\left(N_{2}-1\right)\left(N_{1}+1\right)+2N_{1}-2,\cdots ,\left(N_{2}-1\right)\left(N_{1}+1\right)+N_{1}\}\\ =\{N_{2}\left(N_{1}+1\right)+N_{1}-2,\cdots ,N_{2}\left(N_{1}+1\right)-1\}. \end{array}$$

Based on the above results, we can sum up that the union of $L\cup \mathrm{Diff}\left(L^{3},L^{2}\right)\cup \mathrm{Diff}\left(L^{2},L^{1}\right)\cup \mathrm{Diff}\left(L^{3},L^{1}\right)$ and its counterpart set cover the consecutive integers from $-N_{2}\left(N_{1}+1\right)-N_{1}+2$ to $N_{2}\left(N_{1}+1\right)+N_{1}-2$ with no integer absence, that is to say, the co-array functions like a enlarged ULA. Furthermore, it is easy to verify that the total number of DOFs generated by such virtual array is $2N_{2}\left(N_{1}+1\right)+2N_{1}-3$. This completes the proof.
